# Preparation of CO_2_‑Triggered Extrudable Chitosan for Fat Production

**DOI:** 10.1021/acsbiomaterials.5c01005

**Published:** 2025-07-16

**Authors:** Andrea Fiorati, Beatrice Sottini, Matteo Pavarini, Margherita Pallaoro, Gabriela Graziani, Roberto Casalini, Alessia Di Giancamillo, Luigi De Nardo, Lina Altomare

**Affiliations:** ∇ Department of Chemistry, Materials, and Chemical Engineering “G. Natta”, 18981 Politecnico di Milano, Piazza Leonardo da Vinci 32, I-20133 Milano, Italy; ‡ INSTM, Local Unit at Department of Chemistry, Materials, and Chemical Engineering “G. Natta”, 18981 Politecnico di Milano, Piazza Leonardo da Vinci 32, I-20133 Milano, Italy; § Department of Veterinary Medicine and Animal Sciences (DIVAS), 9304 University of Milan, Via dell’Università 6, 26900 Lodi, Italy; ∥ Department of Biomedical Sciences for Health, University of Milan, Via Mangiagalli 31, 20133 Milan, Italy

**Keywords:** Chitosan, Hydrogels, Acid-free, Food ingredients, Fat mimicry

## Abstract

Cultured ingredients are becoming increasingly important in the food industry. However, reproducing fat ingredients remains a major challenge. Hydrogel scaffolds are commonly used to culture cells for regenerative purposes. Among these, chitosan is a promising material, as it supports high cell viability. Still, its use is limited by conventional processing in acetic acid, whose residuals hamper cell availability and can modify food taste. Here, we propose a methodology for processing chitosan that enables CO_2_-triggered structural modulation, supporting its application as a matrix for cultured food ingredients. For the first time, we add cells to the developed gel by standard mixing and using a newly developed method based on embedding predifferentiated adipocyte pellets. The findings reveal that the gel is stable and results in optimal cell viability when the cells are embedded in differentiated pellets. The system exhibits an effective release of lipids, thus validating its potential for application in edible fat-based ingredients. Moreover, the gel shows an interesting shear-thinning behavior, paving the road for extrusion-based processing.

## Graphical abstract

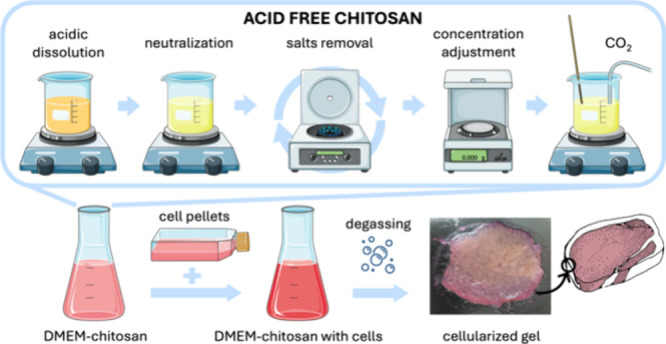

## Introduction

Cultured ingredients are becoming increasingly popular in food technology research, pushed by the need for technical solutions to address the ethical and environmental concerns associated with both extensive and intensive livestock farming. While most research focuses on reproducing muscles, fats are key in determining cultured food’s taste, texture, smell, appearance, and nutritional profile.^1^ However, engineering fats in vitro with desired characteristics presents considerable challenges.^2^ Adipose tissue plays a critical role in metabolic regulation and sensory attributes such as flavor and texture in food. To replicate these properties effectively in vitro, biocompatible scaffolding materials that support adipogenesis while mimicking the native extracellular matrix (ECM) are paramount.^3^ In the context of cultured fat development, the use of edible, plant-based scaffolds has shown encouraging results. For example, sorghum prolamin scaffolds have supported adipocyte growth and contributed to hybrid cultured meat products with enhanced sensory characteristics, underscoring the impact of scaffold composition on final tissue function.^3^ Similarly, rye secalin-derived porous scaffolds have been successfully employed to support porcine adipocyte adhesion and differentiation, demonstrating the feasibility of grain-derived proteins as matrices for engineered fat tissues.^4^

A critical aspect of engineering fat-like tissues is the design of a biocompatible scaffold that promote cell viability, adipogenic differentiation, and triglyceride accumulation *in vitro*.^5,6^ This process typically involves the differentiation of mesenchymal stem cells (MSCs) into preadipocytes, followed by maturation into lipid-laden adipocytes, thus emulating the natural progression of adipose tissue development.^5,6^ Among the various hydrogels proposed for adipose tissue engineering, interesting results in producing fatty derivatives are obtained using collagen or alginate.^5,7,8^

Chitosan stands out as a particularly promising candidate. It is extensively used in edible food packaging and as a functional food ingredient, and it has gained considerable attention in tissue engineering applications, including those aimed at fat tissue mimicry.^9–13^ Notably, in adipose tissue culture systems, chitosan has been reported to inhibit lipid oxidation, a key challenge in maintaining fat tissue stability in vitro.^14^ In addition, it is generally obtained from renewable sources, such as waste shellfish, enhancing the sustainability of the solution.^12^ It can also be obtained by fungi,^15,16^ holding the potential to be used for plant-based ingredients.^17^

Despite these advantages, chitosan applicability in food ingredients is limited by its insolubility in water, necessitating the use of acetic acid for processing.^12^ This can lead to undesirable acid residuals, potentially hampering cell viability and altering the organoleptic characteristics of the final food ingredient.^18^ While methods to neutralize or remove acid exist,^19,20^ an intrinsically acid-free processing route remains preferable.

This work reports a novel procedure to manufacture cell-loaded, acid-free chitosan hydrogels, based on an adaptation of the method proposed by Sakai et al.^21,22^ Instead of using acetic acid solutions, the procedure exploits the formation of carbonic acid (H_2_CO_3_) *in situ* through carbon dioxide (CO_2_) dissolution in water. The pH variation triggers the changing of the gel viscosity, allowing its processing by conventional solvent casting. This characteristic not only is crucial for gentle cell encapsulation and developing fat-like food ingredients but also paves the road for advanced processing techniques such as extrusion. The present study explores the use of chitosan hydrogels as a platform for *in vitro* fat tissue regeneration, focusing on their physicochemical compatibility with adipocyte growth and differentiation. By leveraging chitosan’s modifiable structure and natural affinity for ECM-mimetic interactions, we aim to establish a reproducible, food-safe scaffold conducive to adipose tissue engineering. To this aim, we first tested the chitosan gel potential in encapsulating cells while maintaining their viability. Subsequently, we implemented a novel cell encapsulation approach involving the embedding of preformed pellets of differentiated adipose cells.

## Materials and Methods

Chitosan (CS) powder (75% deacetylated; medium molecular weight; Lot # STBF3507V), glycerol anhydrous >99.0%, hydrochloric acid, glacial acetic acid (AcOH), sodium hydroxide (NaOH), Dulbecco modified eagle’s medium powder (DMEM) with 1000 mg L^–1^ glucose and l-glutamine, without sodium bicarbonate were purchased from Sigma-Aldrich (St Louis, MO, USA). Carbon dioxide (CO_2_, E290, food grade) was bought in a pressurized steel cylinder (P = 6 MPa, T = 28 °C). All reagents were used without further purification. The cell culture tests were conducted using mouse preadipocytes 3T3-L1 cells. Three different culture media were employed: a Complete Medium (CM), a Differentiative Medium (DM), and a Maintenance Medium (MM). CM was prepared, supplementing DMEM with 1 mM sodium pyruvate, 10% fetal bovine serum (FBS), 4 mM l-glutamine, and 1% penicillin-streptomycin. DM refers to High Glucose DMEM enriched with 10% v v^–1^ fetal bovine serum (FBS), 10 mM HEPES, 4 mM l-glutamine, 1 mM sodium pyruvate, 1% penicillin-streptomycin, 1 μg mL^–1^ insulin, 0.5 mM 3-isobutyl-1-methylxanthine (IBMX), 1 μM dexamethasone (DEX), and 2 μM rosiglitazone. MM is composed of High Glucose DMEM with 10% v v^–1^ FBS, 10 mM HEPES, 1 mM sodium pyruvate, 4 mM l-glutamine, 1% v v^–1^ penicillin-streptomycin and 1 μg mL^–1^ insulin.

### Preparation of CO_2_-Chitosan Hydrogels

The acid-free chitosan solution was prepared following a previously reported procedure, schematized as follows (Figure 1):

a) Chitosan (2.5% w v^–1^) was dissolved in an acidic aqueous solution (hydrochloric acid 0.15 M or 0.7–1% acetic acid).
b) The solution was neutralized (pH > 7) by the addition of aqueous NaOH (0.5 M) under magnetic stirring.
c) The obtained hydrogel was repeatedly washed with deionized water by centrifugation (4000 rpm, 3 min) to separate the gel from water until the rinsing water reached a low conductivity (<80 μS cm^–1^). The resulting hydrogel exhibited a pH of approximately 6.9.
d) The final chitosan concentration was gravimetrically determined by drying an aliquot of the hydrogel.
e) CO_2_ gas was bubbled into the hydrogel at atmospheric pressure and cooled in an ice bath under magnetic stirring to dissolve it. The resulting chitosan solution exhibited a pH of approximately 5.1.

**Figure 1:**
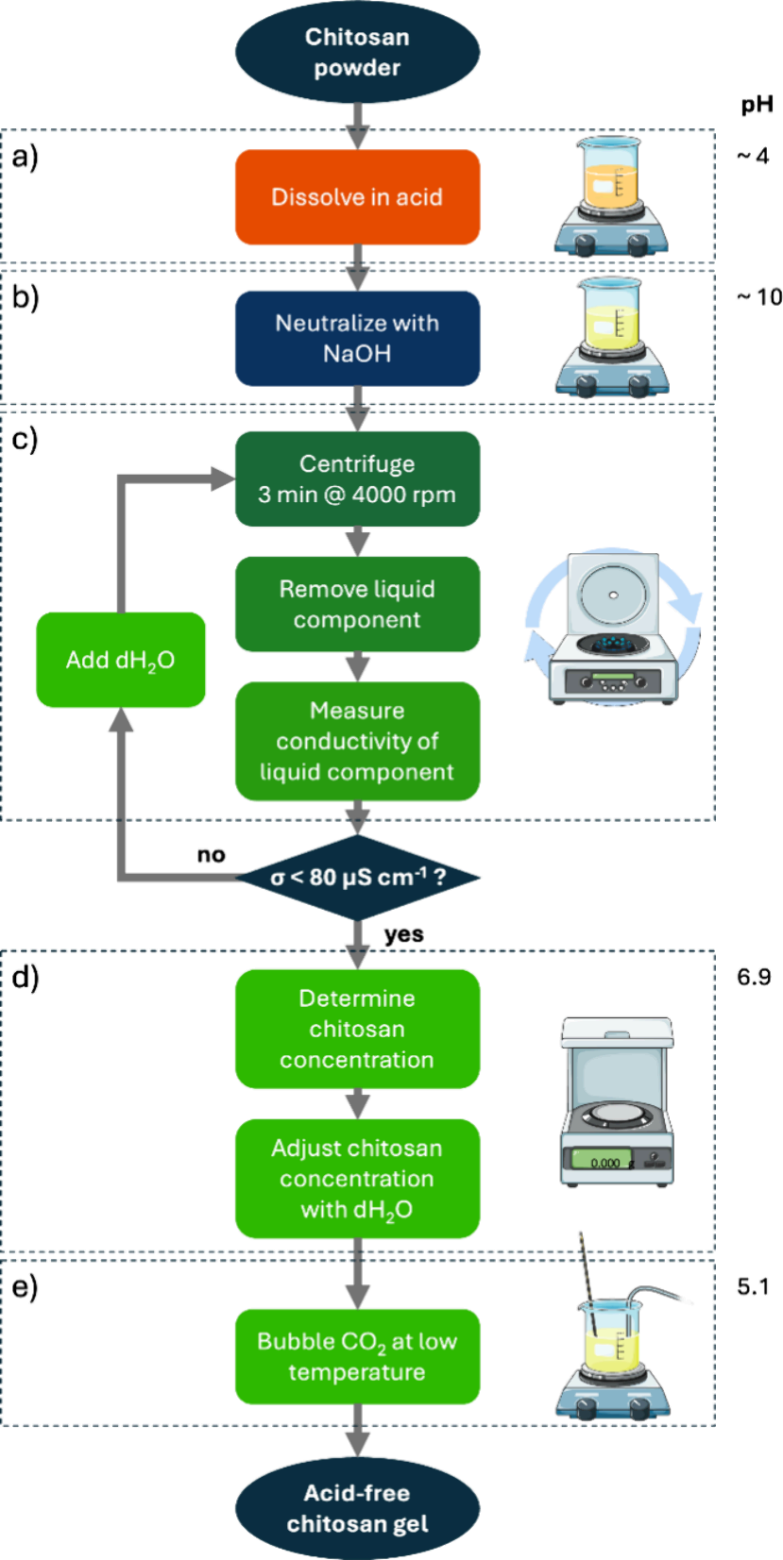
Schematic of the acid-free chitosan gel preparation. The details of blocks a-e are provided in the Materials and Methods section. The color changes show solution pH variations.

CO_2_ molecules react with H_2_O, forming carbonic acid H_2_CO_3_ and lowering the pH value: in this way, amino moieties of chitosan were protonated, promoting chitosan chain dissolution and subsequently increasing the homogeneity of the hydrogel. Deionized water was added stepwise during the hydrogel dissolution to achieve the desired final concentration (1–2%).

### Preparation of Sterile DMEM-Enriched CO_2_-Chitosan Hydrogels

All CO_2_–chitosan solutions used for cell experiments were sterilized by autoclaving (121 °C, 20 min, 2 atm) and subsequently supplemented with powdered DMEM (without sodium bicarbonate). As the autoclaving process removes dissolved CO_2_, a second CO_2_ bubbling step was performed under sterile conditions. To prevent contamination, the CO_2_ gas was filtered through a 0.2 μm membrane prior to bubbling into the chitosan solution. Immediately before use, powdered DMEM (lacking sodium bicarbonate) was added to the solution to support cell viability. When required, cells were introduced into the hydrogel immediately after the DMEM powder had fully dissolved. During experiments, the sterile, DMEM-enriched CO_2_–chitosan solutions were left to rest in air for 20 min to enable CO_2_ release and induce the hardening of the gel. After this resting period, samples were brought into contact with the culture medium. The pH of the culture environment was monitored both visually, through the phenol red indicator present in the culture medium, and by spot-checking with litmus paper. In all cases, the pH remained within the physiological range suitable for cell culture (7.0–7.6).

### Potentiometric Titration and Zeta Potential Measurements

According to literature protocols,^23,24^ 0.1 g of chitosan was dissolved in 10 mL of HCl (0.1 M) and subsequently diluted with 10 mL of deionized water. The resulting solution was titrated by incremental addition of NaOH (0.1 M) under continuous stirring, and the pH variation was recorded by using a pH meter (HI5222, Hanna Instruments) equipped with a glass electrode (model HI1330B, Hanna Instruments). The p*K*_a_ of chitosan was determined from the inflection point of the resulting sigmoidal titration curve, yielding a value of 6.3.

Zeta potential measurements were performed using a Zetasizer Nano ZS (Malvern Panalytical Ltd., 632.8 nm), equipped with a dip cell kit (Malvern Panalytical Ltd.). Prior to each measurement, the pH of the chitosan samples (0.05% w/w) was recorded. Measurements were carried out at 25 °C using the Smoluchowski approximation.^25^ For reproducibility, 100 runs were performed per sample (five measurements of 20 cycles each). When necessary, the pH of the chitosan solutions was adjusted by adding the appropriate volumes of HCl (0.1 M).

### Rheological and Extrusion Test

The rheological characterization of chitosan hydrogels was performed using a rotational rheometer (MCR 302, Anton Paar, Italy) with a parallel plate geometry (Ø = 25 mm); the gap was set at 1 mm and temperature was set at 23 °C. The linear viscoelastic region curves of chitosan hydrogels and CO_2_-chitosan hydrogels were obtained through amplitude sweep test, by applying a shear strain in the range between 0.01 and 500%. The oscillation frequency was maintained at 1 Hz during all the experiments. The viscosity (η) vs shear rate (*γ̇*) curves were obtained after a preconditioning phase (shear rate = 1000 s^–1^ for 10 s, then shear rate = 0.1 s^–1^ for 50 s) by applying a shear rate in the range 0.1–1000 s^–1^. Viscosity behavior was studied before and after the addition of CO_2_.

Three-step oscillatory shear tests were conducted on both chitosan and CO_2_-chitosan hydrogels to assess their shape recovery behavior following exposure to high shear strain, simulating conditions relevant to extrusion-based printing. The testing protocol consisted of an initial low strain phase at 0.5% shear strain for 2 min, followed by a high strain phase at 500% for 3 min, and a recovery phase at 0.5% shear strain for 40 min. Throughout all phases, the oscillation frequency was maintained at 1 Hz. Storage (G′) and loss (G″) moduli were continuously recorded to monitor the structural recovery. All measurements were performed in triplicate.

Extrusion tests were performed using a custom-designed setup coupled with a uniaxial tensile testing machine (MTS 1/MH, 5 kN load cell, MTS Systems, Eden Prairie, MN, USA) operated in compression mode. The setup included a perforated poly­(methyl methacrylate) (PMMA) cylinder that securely held a 5 mL syringe in a vertical position, ensuring proper alignment with the machine plate. A container was positioned beneath the syringe needle (either 18G or 22G) to collect the extruded hydrogel.^26^

Hydrogels were loaded into sterile 5 mL syringes and extruded at room temperature (23 °C). The plate was initially lowered at a speed of 1 mm min^–1^ until a contact force of 0.15 N was detected, confirming engagement with the syringe plunger. Subsequently, extrusion was performed at a speed of 9 mm min^–1^ (corresponding to an approximate flow rate of 1 mL min^–1^) over a displacement of 9 mm, while the required force was continuously recorded.

Prior to testing, all hydrogel samples were homogenized by transferring them back and forth between two syringes connected via a three-way Luer stopcock, simulating the cell-loading procedure described later. For CO_2_–chitosan hydrogels, CO_2_ was introduced before the homogenization step.

### Degradation Test

The degradation tests were conducted by dipping the CO_2_-chitosan samples (500 μL) in 2.5 mL of DMEM enriched with antibiotics (penicillin and streptomycin 1% w w^–1^) and 0.02% (w w^–1^) sodium azide (NaN_3_) and then incubating them at 37 °C. The culture medium was removed at each time point, and the samples were weighed at progressive time steps. Then, they were covered with 2.5 mL of fresh culture medium and stored again in the incubator.

The residual hydrogel weight (*RW*_*%*_) was calculated as in eq 1:

1 $${RW}_{\%}=\frac{w_{f}-w_{i}}{w_{i}}\times 100$$

*w*_*f*_ is the sample’s weight after the medium is removed, and *w*_*i*_ is the sample’s initial weight before soaking in the culture medium.

### Cytotoxicity Assay

An indirect cytotoxicity test was carried out *in vitro* according to EN ISO 10993-5.^40^ Preadipocytes 3T3-L1 cells were seeded in 96-well tissue culture polystyrene plates (1 × 10^4^ cells per well) and cultured in Complete Medium at 37 °C (5% CO_2_ and humidified atmosphere) until 70% confluence was reached. Culture medium eluates were prepared by exposing CM (1 mL) to a sterile DMEM-enriched CO_2_-chitosan sample (200 μL). Sterile DMEM-enriched CO_2_–chitosan solutions were freshly prepared prior to each experiment, as described above.

Three time-points were selected: 1, 3, and 7 days (n = 3). Cells were cultured for 24 h with either eluates or fresh culture medium. Cell viability was assessed by Alamar blue assay, and fluorescence was measured by a Synergy H1 spectrophotometer (BioTek, Rodano, Italy; λ_exc_ = 540 nm, λ_em_ = 595 nm). The percentage cell viability was calculated according to eq 2:

2 $$Cell\;viability\;\left[\%\right]=\frac{f_{eluates}-f_{AlamarBlue}}{f_{control}-f_{AlamarBlue}}\times 100$$

where *f* is the fluorescence value of eluates, control, and Alamar Blue reference.

### Direct Cytocompatibility Assay

DMEM powder was reconstituted by dissolving it in the CO_2_-chitosan hydrogel in order to achieve the proper culture medium concentration. 3T3-L1 preadipocyte cells were suspended in a FBS (F7524, Sigma-Merk) solution and mixed with the sterile DMEM-enriched CO_2_-chitosan after loading the two components into two sterile syringes connected using a three-way Luer Stopcock. For the sake of example, to prepare 4 samples, 3 × 10^5^ cells, suspended in 200 μL of FBS solution, were mixed with 1 mL of DMEM-enriched CO2-chitosan. The loading was performed by gently mixing the contents of the two syringes until the cells were evenly distributed within the gel. Subsequently, 200 μL of the cell-laden hydrogel was extruded into each well of a 24-well culture plate and left to rest for 20 min under sterile conditions to allow CO_2_ to escape from the hydrogel, promoting its transition to a more solid-like state. After this resting period, 1.5 mL of culture medium was added to each well, and the samples were incubated at 37 °C. Cell viability was assessed up to 7 days by Alamar blue assay. After 7 days, the samples were transferred to a new multiwell culture plate, and viability was assessed again after 7 + 1 and 7 + 3 days, to evaluate if cells inside the hydrogels are still viable.

### Live Dead Assay

Ten days after seeding, a LIVE/DEAD assay was performed on seeded scaffolds (n = 3) to observe cell viability qualitatively and quantitatively. Samples were incubated in the staining solution (10 μM AM Calcein and 2 μM propidium iodide in PreDMEM without FBS) for 40 min, washed three times with PBS, and immersed in CM without FBS. Images were acquired using an Olympus BX51WI fluorescence optical microscope at 200× and 400× magnifications and processed with ImageJ Fiji software (NIH, United States).

### Adipocyte Pellet Incorporation

Preadipocyte 3T3-L1 was seeded 1 × 10^4^ cells cm^–2^ in CM (T175). When cells reached 70% confluence, the medium was substituted with a differentiation medium (DM) to promote adipocyte development. Four days after culturing in the DM, cells were detached in pellets (35–40 mg each, corresponding to about 4.2 × 10^6^ cells per pellets). From each T175 flask, 4 pellets were isolated and suspended in 500 μL of sterile DMEM-enriched CO_2_-chitosan hydrogel without pellet disaggregation. Sterile DMEM-enriched CO_2_-chitosan solutions were freshly prepared prior to each experiment, as described above.

The medium was changed every 3 days, alternating between DM and MM, according to the literature.^27–29^ Alamar blue viability test and Oil Red O staining were performed after 6, 9, and 13 days of culture in the CO_2_-chitosan hydrogel.

### Oil Red O Staining

In whole-mount samples, lipid droplets in 3T3-L1 cells were stained by the Oil Red O staining method.^30^ Oil Red O staining solution was prepared by dissolving 300 mg of Oil Red O powder in 100 mL of isopropanol and then diluted 2:3 with distilled water and filtered. Briefly, cells were fixed with 4% paraformaldehyde for 30 min and then washed with distilled water. Subsequently, the samples were incubated into staining solution for 1 h. Finally, samples were washed in distilled water and incubated in a DAPI Vetashield antifade mounting medium (Vector laboratories, Newark, USA) for 30 min in the dark to stain cell nuclei properly. The microscopical examination was performed with an Olympus BX51WI fluorescence optical microscope at 200× and 400× magnifications. The content of stained lipid droplets has been semiquantitatively evaluated by spectrophotometry, as reported in the literature.^31^ The stained samples were immersed overnight in isopropanol to dissolve the Oil Red O stained lipid droplets. Then, the extracted dye absorbances were measured at 510 nm. Moreover, the cumulative stained lipid droplet extension was quantified by Oil Red O signal distribution on fluorescence images (referring to at least 3 samples for each time point) after proper thresholding and compared to the corresponding DAPI signal. The value was expressed as the lipid/nuclei normalized surface ratio.^32^

## Results and Discussion

### Preparation of CO_2_-Chitosan Hydrogels

The flux diagram in Figure 1 summarizes the proposed methodology for preparing acid-free chitosan hydrogels as 3D gel-embedded culture media for fatty cells. Chitosan was initially dissolved in an acidic aqueous environment (Figure 1a): preliminary process optimization indicated that acetic acid solutions are more suitable than aqueous HCl for chitosan dissolution, providing a faster and more practical process. Due to subsequent washing steps (Figure 1c), the final CO_2_-chitosan solutions are not affected by the choice of acid used in the initial step. All samples described in this study were prepared by using aqueous acetic acid solutions.

The hydrogel obtained after the washing steps (Figure S1b) exhibited a pH of approximately 6.9. Bubbling CO_2_ into the hydrogel (20 mL) after cooling resulted in a pH reduction to around 5.1 within approximately 1 h. Extending the CO_2_ bubbling time beyond this point did not lead to a further pH decrease (Figure S2). In some cases, the total bubbling time depends on the total volume of chitosan. Interestingly, the use of a domestic benchtop apparatus designed for carbonating water proved to be effective for introducing CO_2_ into chitosan hydrogels. These devices operate at slightly above atmospheric pressure, which helps reduce carbonation time—particularly when handling larger volumes of chitosan solution. However, the final pH achieved using this method was comparable to that obtained by CO_2_ bubbling at atmospheric pressure. While this approach is valuable for scaling up material production, maintaining sterile conditions with such equipment is challenging. Therefore, all samples prepared for this study were produced by bubbling CO_2_ at atmospheric pressure.

The addition of CO_2_ to water leads to the *in situ* formation of carbonic acid, which effectively induces protonation of chitosan moieties.^11,21,22^ To validate this mechanism, we measured the zeta potential and pH of diluted chitosan solutions before and immediately after CO_2_ addition (Figure 1d and e) and compared the results with those obtained from chitosan solutions whose pH was adjusted using HCl (Figure 2). The zeta potential of the diluted chitosan solution, following the previously described washing step and prior to any acidification, was approximately +17 mV, with a pH of around 6.9—corresponding to about 20% protonation of the amino groups. Upon CO_2_ addition (Figure 1e), the pH decreased to approximately 5.1, accompanied by an increase in zeta potential to around +31 mV, confirming the protonation of the chitosan chains. At this pH, the estimated extent of protonation was approximately 94%. This result aligns well with the zeta potential measured in chitosan solutions acidified to the same pH using HCl, which reached about +38 mV. The observed difference in zeta potential between the two conditions can be attributed to the different ionic strength generated by the counterions surrounding the protonated chitosan molecules.^33^ The extent of amino group protonation (%) was calculated using the Henderson–Hasselbalch equation,^34^ with a p*K*_a_ value of 6.3 for the R–NH_3_^+^/R–NH_2_ dissociation pair, as determined by potentiometric titration of chitosan (Figure S3).

**Figure 2:**
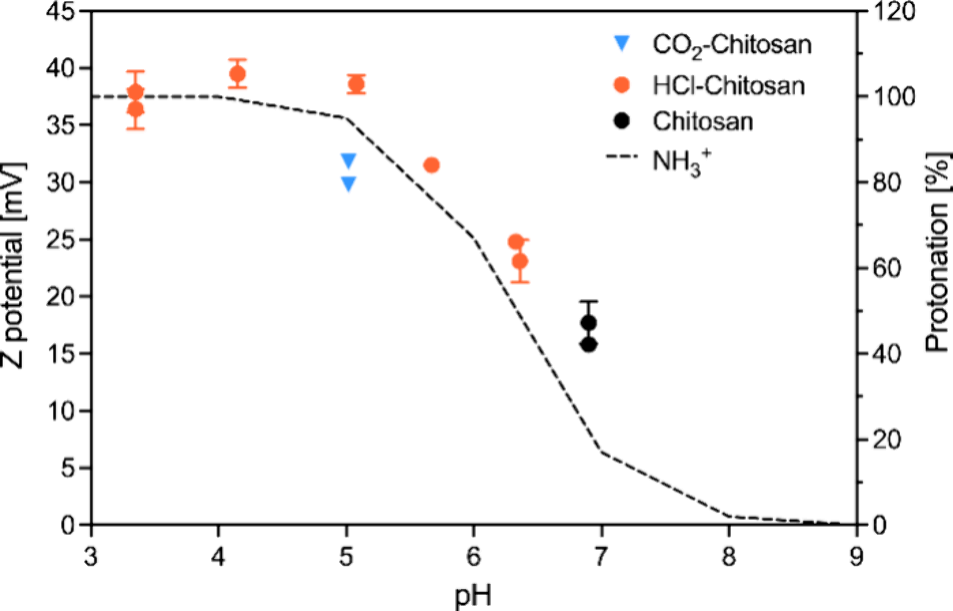
Zeta potential curves of chitosan (0.05% w/w) at different pH values. Black circles represent samples before any addition of CO_2_ or HCl; orange circles represent samples after pH modification with HCl; and light blue circles indicate samples acidified by CO_2_ addition. The dashed line (corresponding to the secondary axis) represents the calculated extent of protonation.

While the *in situ* formation of carbonic acid can be exploited to modulate the softening of chitosan hydrogels, the reversible nature of this process—stemming from the rapid decomposition of carbonic acid into water and carbon dioxide—can also be leveraged to restore the hydrogel’s structural integrity. Specifically, exposing CO_2_-saturated chitosan solutions to air enables the efficient removal of carbonic acid, resulting in the rapid formation of compact hydrogels. Since CO_2_ release occurs faster than water evaporation, the hydrogel composition remains largely unchanged after the transition. This reversible behavior offers a convenient method for modulating the viscoelastic properties of chitosan hydrogels while avoiding residual acidic byproducts, one of the main limitations of traditional chitosan dissolution methods. This behavior was recently exploited by our group to produce homogeneous chitosan films for food coating applications.^11^

### Rheological Characterization

Figure 3 shows the viscosity vs shear rate curves of the tested specimens. All tested conditions exhibited shear-thinning behavior, as evidenced by a decrease in viscosity with increasing shear rate (Figure 3a, c, and e). As expected, the presence of CO_2_ leads to significantly lower values of viscosity: the absence of CO_2_ is related to deprotonation of chitosan amino groups, which causes a more compact hydrogel, more hydrophobic interactions between chitosan chains, and thus a higher viscosity level.^21,22,35^

**Figure 3:**
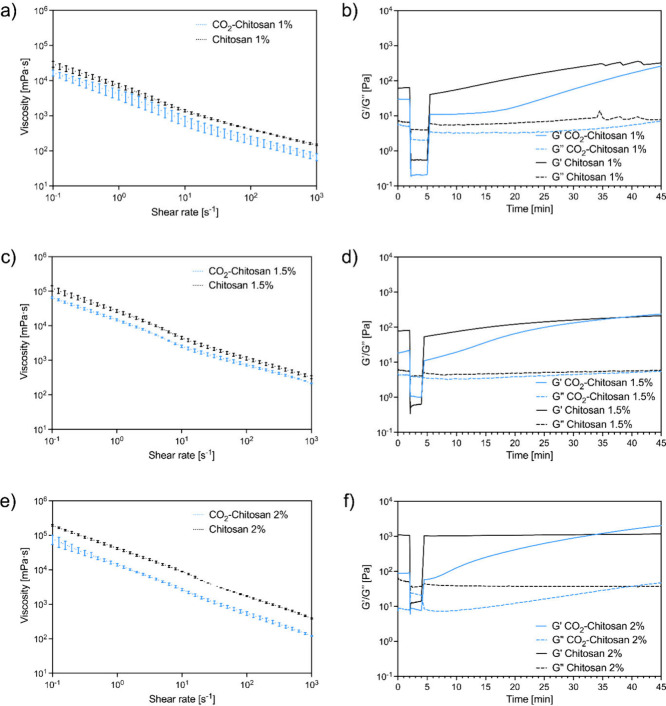
Viscosity vs shear rate curves of acid-free chitosan hydrogels before and after CO_2_ addition: a) 1% w/v, c) 1.5% w/v, e) 2% w/v; three-step oscillatory shear tests before and after CO_2_ addition: b) 1% w/v, d) 1.5% w/v, f) 2% w/v.

Amplitude sweep experiments revealed a significant reduction in the storage modulus (G′) of the hydrogel upon CO_2_ addition (Figure S4), with values decreasing by up to 1 order of magnitude. As expected, decreasing the chitosan concentration led to a corresponding decrease in both the storage (G′) and loss (G″) moduli. However, in all tested conditions, G′ remained consistently higher than G″, even after CO_2_ treatment. This indicates that CO_2_–chitosan hydrogels maintain a predominantly elastic behavior and cannot be classified as true solutions, despite their ability to flow under gravity, as observed in the tube inversion test (Figure S1). To the best of our knowledge, this is the first report in the literature detailing the storage and loss moduli of CO_2_–chitosan dispersions. Previous studies have typically described such systems as solutions.^11,21,22^ We attribute this discrepancy to variations in the chitosan source, particularly in the molecular weight and degree of deacetylation, which are known to vary between batches.

According to the shear rate range values during ink extrusion reported in the literature,^36^ these results reflect a potential extrudability and printability of acid-free CO_2_-chitosan hydrogels. Indeed, the shear-thinning behavior of a biomaterial ink is a crucial property in the 3D (bio) printing technique: hydrogel viscosity has to allow its extrusion, preventing needle clogging and a good recovery once the shear is removed (after the printing process).^37^ As reported in ref 36, the shear rate experienced by the ink during extrusion is dependent on its viscosity and can be calculated using the following equation (eq 3):

3 $$\mathrm{\gamma ̇}=\frac{4Q}{\pi R^{3}}\frac{3n+1}{4n}$$

with *Q* = flow rate, *R* = needle inner radius, and (*n* – 1) = the slope of viscosity vs shear rate graph on a log–log plot.

Compared to a standard solution of chitosan in acetic acid, the viscosity values of our acid-free hydrogels possess lower viscosity (up to 1 order of magnitude) at high shear rate and higher viscosity at low shear rate (again up to 1 order of magnitude).

To support the potential use of CO_2_–chitosan hydrogels as (bio)­inks for 3D printing applications, we conducted preliminary evaluations of their elastic recovery following exposure to high shear stress through three-step oscillatory rheological tests (Figure 3b, d, and f). These measurements revealed that the storage modulus (G′) recovered almost immediately after shear cessation in both the CO_2_-free and the CO_2_–chitosan hydrogels. Notably, in the case of CO_2_–chitosan samples, G′ gradually increased, reaching values comparable to those of CO_2_-free hydrogels within approximately 25–30 min. This delayed recovery is attributed to the gradual shift in the H_2_CO_3_–CO_2_ equilibrium due to CO_2_ outgassing. Water loss was considered negligible, as the samples were maintained at ∼100% relative humidity throughout the experiments. The estimated CO_2_ evaporation time thus represents the time scale required for significant changes in the viscoelastic properties of CO_2_–chitosan hydrogels, resulting in a more compact material better suited for handling during cell-embedding procedures.

To further assess the extrudability of CO_2_–chitosan hydrogels, extrusion tests were conducted using two syringe needle sizes (18G and 22G), measuring the force required to extrude 1 mL of hydrogel over 1 min (Figure S5). In the case of non-CO_2_-added hydrogels, the extrusion force profiles were irregular and exhibited sudden spikes, indicating needle clogging—even with the larger 18G needle—resulting in nonuniform extrusion. In contrast, the CO_2_–chitosan hydrogels displayed lower and more consistent extrusion forces, approximately 10 N for 18G and 20 N for 22G needles, suggesting smoother flow and supporting their suitability as printable materials. These findings indicate that CO_2_–chitosan hydrogels may serve as promising bioinks, offering lower shear stress during extrusion—thus potentially reducing cellular damage^37^—while maintaining sufficient viscosity at low shear rates to ensure postprinting shape fidelity, structural stability, and cohesiveness. Nevertheless, given the heterogeneous nature of these hydrogels, further 3D printing experiments are required to confirm and expand upon these preliminary results.

Additionally, the reversible softening and subsequent stiffening of CO_2_-chitosan hydrogels—triggered by the removal of carbon dioxide upon exposure to air—support their potential application as a dynamic matrix for three-dimensional cell culture.

### Degradation Tests

Degradation tests on specimens exposed to a culture medium and incubated at 37 °C were first carried out (Figure 4). When the chitosan concentration in the hydrogel is lower than 2%, the gels face rapid disaggregation in less than 24 h. CO_2_–Chitosan 2% samples experience a steep weight loss (about 35% of initial weight) at early time points, especially on day 1 and up to day 2. Subsequently, the gel remains stable, and no weight variations are experienced for up to 9 days, although an increasing trend after day 8 might indicate further swelling at longer time points. The initial weight loss is compatible with literature data on chitosan gel degradation in PBS and in simulated physiological conditions,^38,39^ that indicate a weight loss between 30 and 50% due to hydrolytic degradation in a concentration-dependent manner.

**Figure 4:**
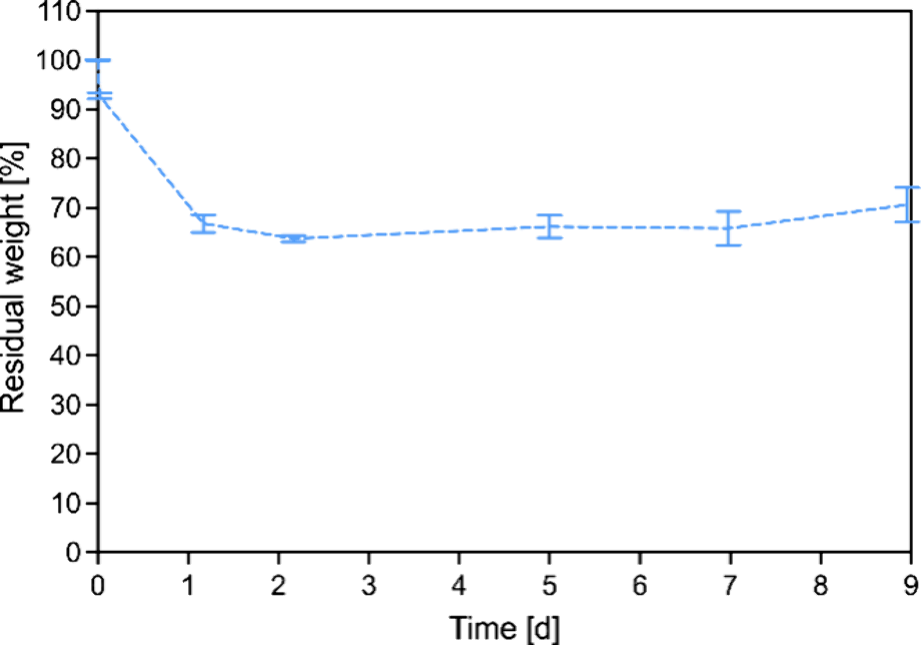
Results of the degradation test on CO_2_–Chitosan 2% hydrogels exposed in DMEM culture medium at 37 °C.

The viscoelastic behavior of the hydrogels during the degradation test was consistent with the residual weight data (Figure S6). Specifically, the storage modulus (G′) measured at the beginning of the experiment was approximately 10^2^ Pa, increased to around 10^3^ Pa after 1 day, and remained stable at subsequent time points.

### Cytotoxicity Assay

For the 2% samples, indirect cytotoxicity tests conducted on conditioned media collected after 1, 3, and 7 days revealed good cell viability after 24 h of exposure, with values of 97.8  ±  9.9%, 102.5  ±  4.7%, and 121.4  ±  4.4%, respectively (calculated using eq 2). All values exceeded the 70% viability threshold defined by ISO 10993-5, confirming the absence of cytotoxic effects.^40^ On the contrary, samples with chitosan concentrations lower than 2% were excluded from cellular tests due to the noticeable hydrogel disaggregation.

### Direct Cytocompatibility Assays

Figure 5a and b report the results of direct cytocompatibility tests. All samples show an increasing trend in cell proliferation between Days 1 and 3, with the 2% chitosan sample also exhibiting continued growth up to Day 7, indicating its potential suitability for the intended application (Figure 5a). To confirm that the metabolic activity observed in the 2% sample at Day 7 was primarily due to cells residing within the hydrogel, the gels were carefully transferred to new multiwell plates. Cell viability was then assessed after an additional 1 and 3 days (i.e., Days 7 + 1 and 7 + 3, respectively, Figure 5b).

**Figure 5:**
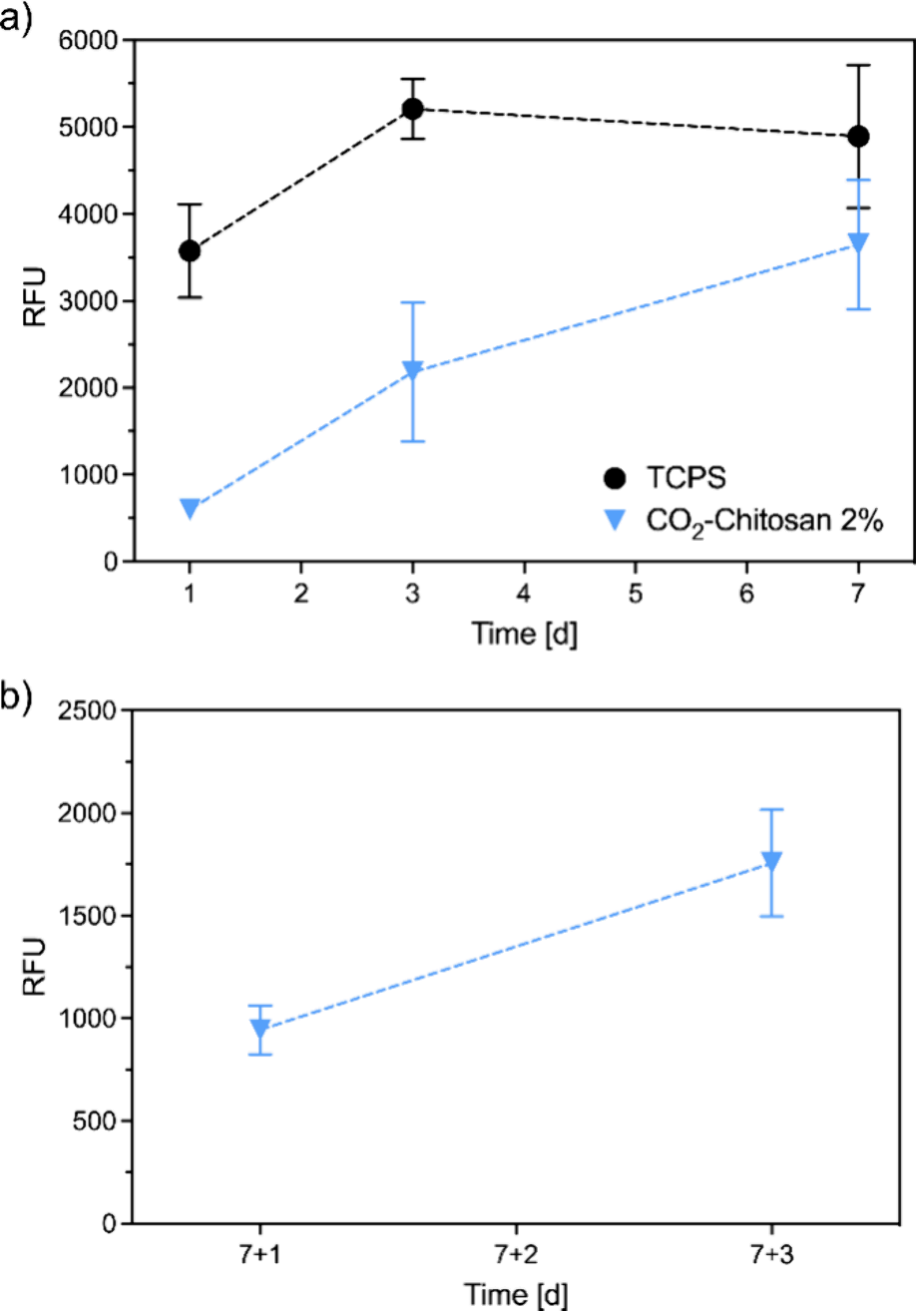
a) Direct cytotoxicity after 1, 3, and 7 days, comparison between CO_2_-chitosan samples and tissue culture polystyrene (TCPS); b) Direct viability at 7 + 1 and 7 + 3 days, after the transfer to new multiwell plates. Data are presented as relative fluorescence units (RFU).

As shown in Figure 5b, an increasing trend in metabolic activity was again observed. The lower absolute values compared to Day 7 in Figure 5a are attributed to the absence of cells that had previously migrated to and proliferated at the well bottom. Notably, no further cell migration was observed after the transfer, supporting the conclusion that the viability signals originated from cells embedded within the hydrogel. A live/dead assay at 10 (7 + 3) days shows a high percentage of live cells (93 ± 8%), with a negligible number of dead ones (Figure 6).

**Figure 6:**
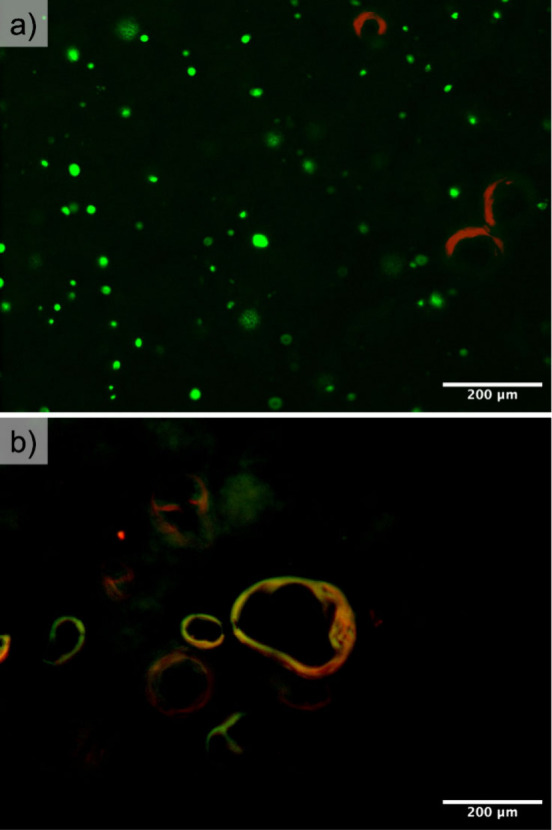
a) Representative micrograph of a live/dead assay in a chitosan sample after 7 + 3 days. Green spots indicate living cells stained with calcein, while red spots represent dead cells stained with propidium iodide. The three circular red marks are artifacts caused by air bubbles trapped in the gel, which were also observed in the control samples without cells; b) live/dead assay of a CO_2_–chitosan control sample after 7 + 3 days, highlighting artifacts caused by air bubbles trapped within the gel.

To explore the ability of the gel to support differentiated cells, 3T3-L1 preadipocytes were first cultured and induced to differentiate in standard culture flasks. Upon the onset of differentiation, the cells were collected as pellets and embedded into the chitosan gel without prior homogenization. This approach was intentionally chosen to promote the formation of 3D cellular aggregates, mimicking native adipose tissue architecture rather than achieving a homogeneous cell distribution, as also reported by other authors.^8^ The viability of the differentiated 3T3-L1 pellets was assessed at days 6, 9, and 13 (Figure 7a). Consistent cell viability was observed throughout the time points, remaining stable from day 6 to day 13, which suggests successful differentiation. The decrease in Alamar Blue relative fluorescence unit (RFU) values between days 6 and 9 was attributed to the alternation between differentiation medium and maintenance medium, a trend that was also observed in the 2D control (Figure S7).

**Figure 7:**
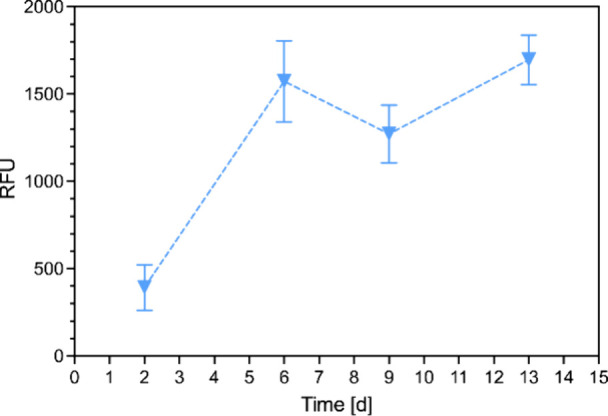
Direct viability at 6, 9, and 13 days for pellets loaded into 2% CO_2_-chitosan hydrogels, measured as relative fluorescence units (RFU).

Oil Red O staining confirmed both the stage of differentiation and the maintenance of the adipogenic phenotype within the gel. Microscopic evaluation revealed a positive Oil Red O staining signal in samples collected at days 6, 9, and 13 of differentiation, indicating the presence of intracellular lipid droplets. The intensity and abundance of red-stained lipid droplets increased over time, suggesting progressive lipid accumulation during the differentiation process (Figure 8). These results support the ability of our hydrogel system to effectively sustain and promote adipogenic differentiation. Semiquantitative evaluation of intracellular lipid accumulation was performed using two complementary approaches to monitor lipid content over time. In the first method, Oil Red O (ORO)-stained lipids were extracted with isopropanol, and absorbance at 510 nm was measured (Figure 9a).^31^ In the second method, the ORO-lipid complexes were quantified through fluorescence microscopy, following established protocols from the literature (Figure 9b).^32^ Both analyses showed a time-dependent increase in the lipid content, corroborating the visual evidence of lipid accumulation observed in the microscopy images (Figure 8).

**Figure 8:**
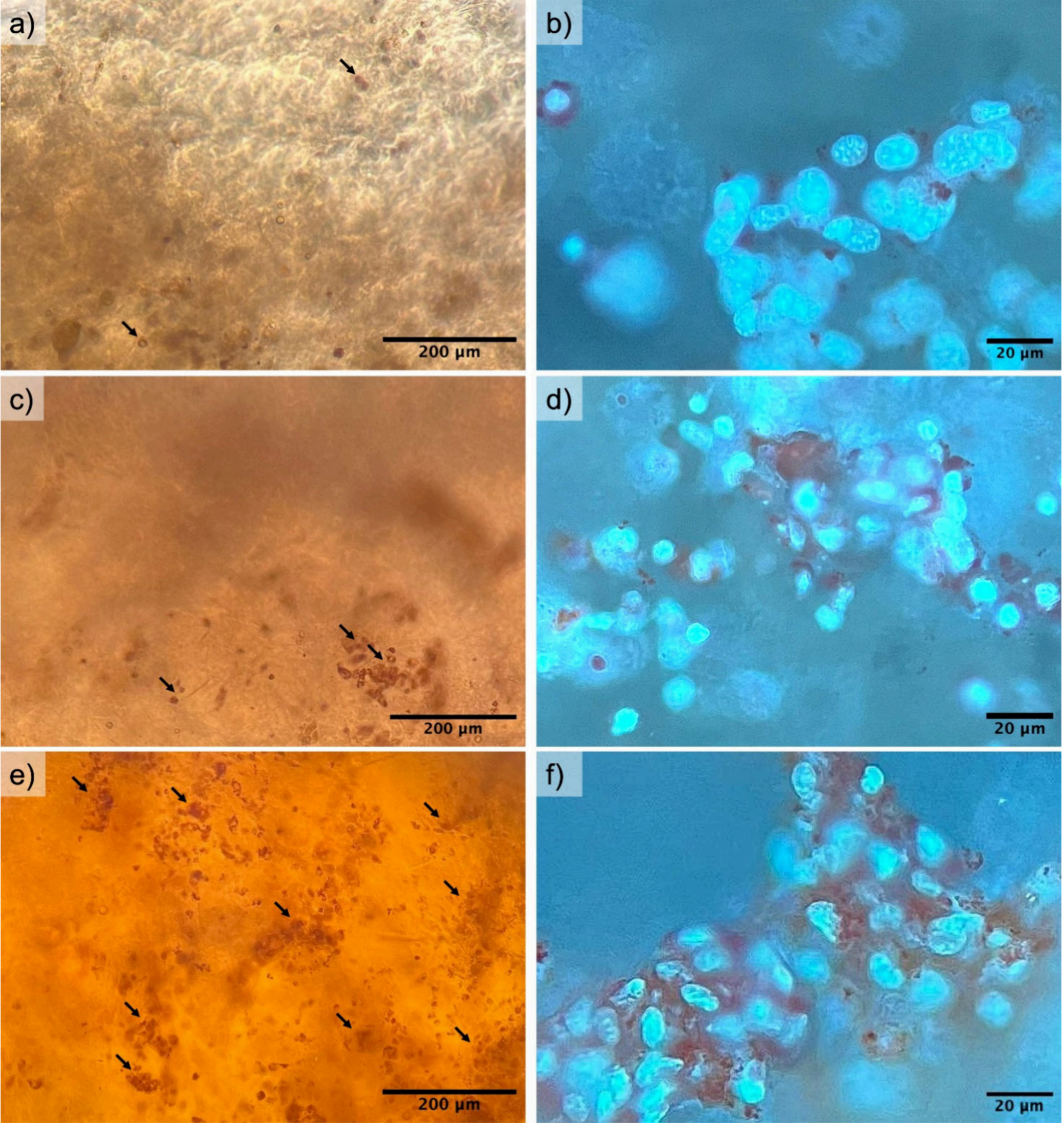
Optical microscopy 40× (left) and 200× (right) fluorescence microscopy 200× (right) images of Oil Red O (ORO)-stained samples at different time points (red = ORO, blue = DAPI). Black arrows indicate lipid droplets stained with ORO. a,b) 6-day differentiation; c,d) 9-day differentiation; e,f) 13-day differentiation.

**Figure 9:**
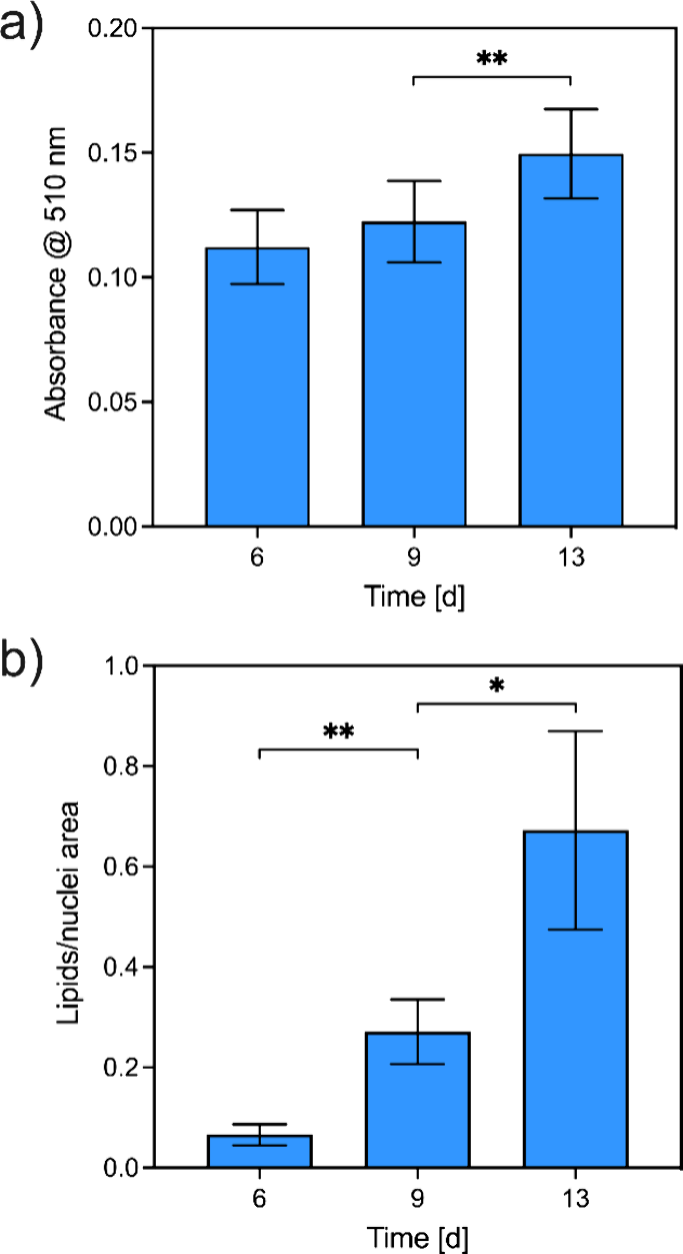
Semiquantitative evaluation of intracellular lipid content by Oil Red O staining (ORO). a) Lipids were extracted with isopropanol, and absorbance at 510 nm was measured to indirectly quantify lipid. b) Quantification of lipid-containing pellet regions relative area from the ORO signal in fluorescence microscopy images (see Figure 8). * *p* < 0.05, ** *p* < 0.005.

Significant limitation in producing cell-cultured fat tissue for food applications often lies in the scalability of the reported processes. Yuen et al. recently introduced an innovative approach to producing bulk cell-cultured fat tissue to address these challenges. Initially, adipocytes are cultured in a 2D environment and then harvested, aggregated into pellets, and subsequently embedded into a 3D construct using binders such as alginate or transglutaminase.^8^ One example of adipogenic differentiation and maturation in three-dimensional matrices made from alginate and collagen is reported by Zagury and colleagues.^5^

Our preliminary findings indicate that CO_2_-chitosan hydrogels support the three-dimensional organization and adipogenic differentiation of 3T3-L1 cells, underscoring their potential as a biomimetic matrix for adipose tissue engineering. These results demonstrate the feasibility of employing CO_2_-induced chitosan hydrogels as a culture platform for adipogenic cells and suggest their applicability in the development of structured lipid-based food components. It is important to emphasize that this study utilized the 3T3-L1 murine preadipocyte cell line, a well-established in vitro model for investigating adipogenesis and metabolic function. While this enabled initial system optimization, future studies involving primary cells derived from livestock species intended for human consumption will be essential to validate the suitability of this platform for producing edible cultured fat.

## Conclusions

This study demonstrates a convenient and effective approach for processing chitosan gels that are capable of incorporating cells while preserving their viability and differentiative potential. The concept was explored using 3T3-L1 preadipocytes as a cell model, resulting in favorable viability and sustained maintenance of their differentiated state within the gel. Despite utilizing murine cell lines, this investigation paves the way for advancing novel ingredients for cultured meat, specifically focusing on the fat component. The resulting scaffolds offer a compelling combination of sustainability, cost-effectiveness, and food safety, alongside a supportive tissue-like structure and appropriate physicochemical properties for adipogenesis. Thus, these chitosan-based scaffolds represent a promising platform for proposed food technology applications.

## Supplementary Material



## Notes

pH variation with CO_2_ bubbling time, potentiometric titration results, rheological characterization, extrudability tests, and photographs of chitosan hydrogels (PDF)
