# Supplementary material for: Preparation of CO2‑Triggered Extrudable Chitosan for Fat Production
Source: ACS Biomater Sci Eng. 2025 Jul 16;11(8):4714–24. doi: 10.1021/acsbiomaterials.5c01005 (PMC12344636; doi:10.1021/acsbiomaterials.5c01005)
Supplement: Supplementary file 1 [file ab5c01005_si_001.pdf]

Supporting information for:
Preparation of CO_2-Triggered Extrudable Chitosan for Fat Production

Andrea Fiorati,[†, ‡,*] Beatrice Sottini,[†,*] Matteo Pavarini,[†, ‡] Margherita Pallaoro,[§] Gabriela Graziani,[†] Roberto Casalini,[†] Alessia Di Giancamillo,[≀] Luigi De Nardo,[†, ‡] and Lina Altomare.[†, ‡]

† Department of Chemistry, Materials, and Chemical Engineering “G. Natta”, Politecnico di Milano, Piazza Leonardo da Vinci 32, I-20133 Milano, Italy.

‡ INSTM, Local Unit at Department of Chemistry, Materials, and Chemical Engineering “G. Natta” Politecnico di Milano, Piazza Leonardo da Vinci 32, I-20133 Milano, Italy.

§ Department of Veterinary Medicine and Animal Sciences (DIVAS), University of Milan, Via dell'Università 6, 26900 Lodi, Italy.

≀ Department of Biomedical Sciences for Health, University of Milan, Via Mangiagalli 31, 20133 Milan, Italy.

* These authors contributed equally to this work.

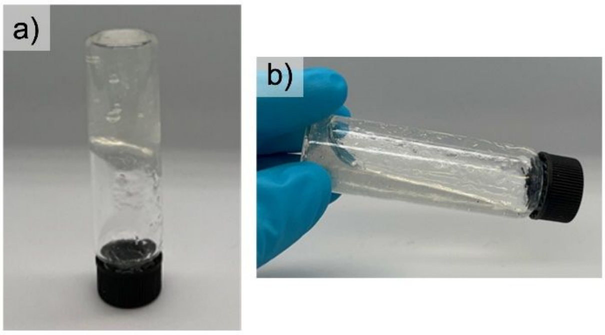

Figure S1. a) Chitosan hydrogel after the washing steps; b) CO_2-chitosan solution obtained by the bubbling of CO_2 into chitosan hydrogel.

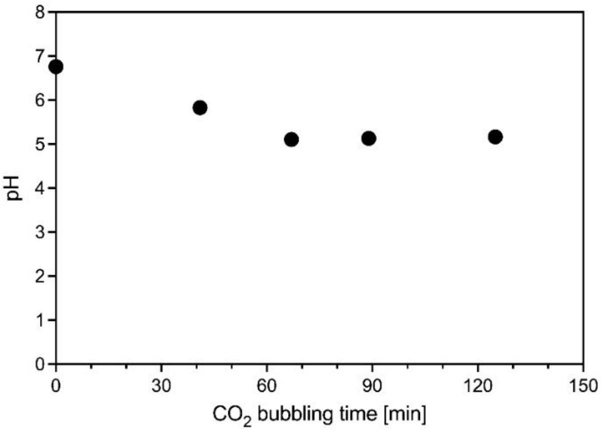

Figure S2. pH variation of a 20 mL chitosan solution (2% w/w) as a function of CO_2 bubbling time.

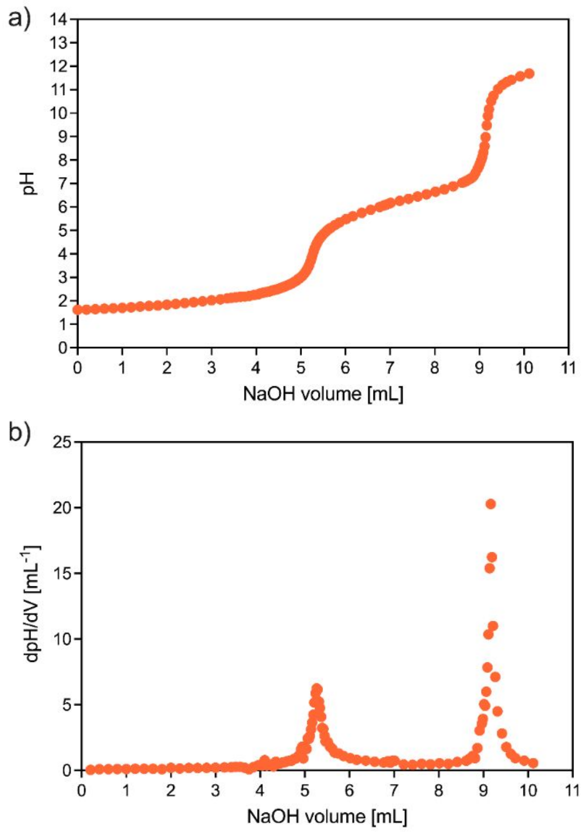

Figure S3. Potentiometric titration curve of 100 mg of chitosan dissolved in 0.1 M HCl (a) and its derivative (b).

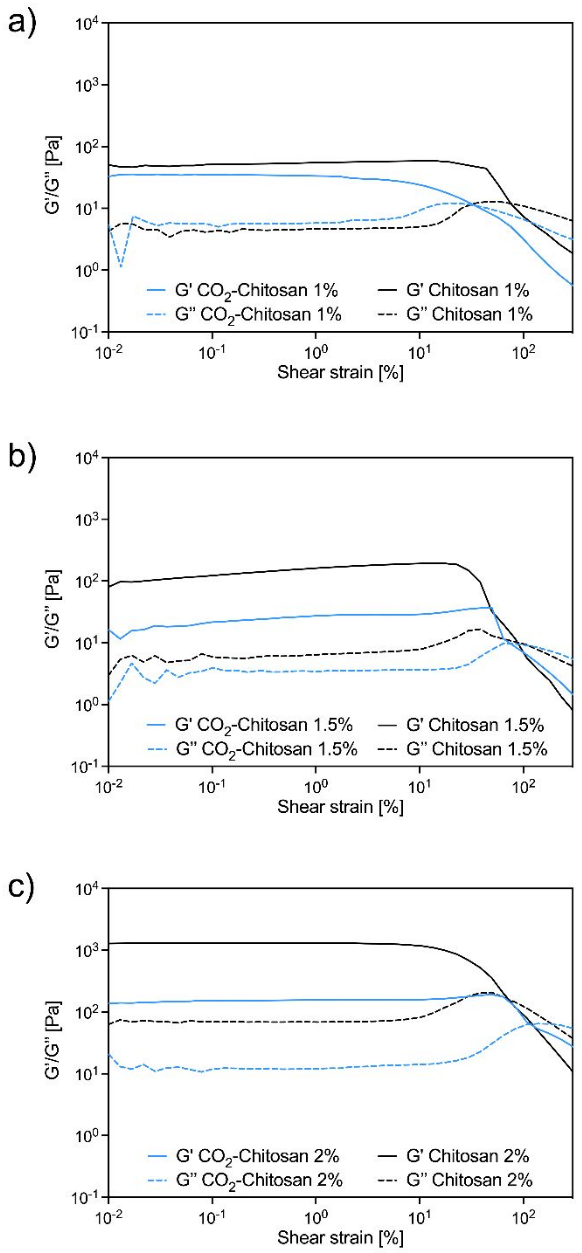

Figure S4. Linear viscoelastic region (LVER) determination of chitosan and CO_2-chitosan hydrogels via amplitude sweep tests. Shear strain was applied in the range of 0.01–500% at a constant oscillation frequency of 1 Hz and temperature of 23 °C.

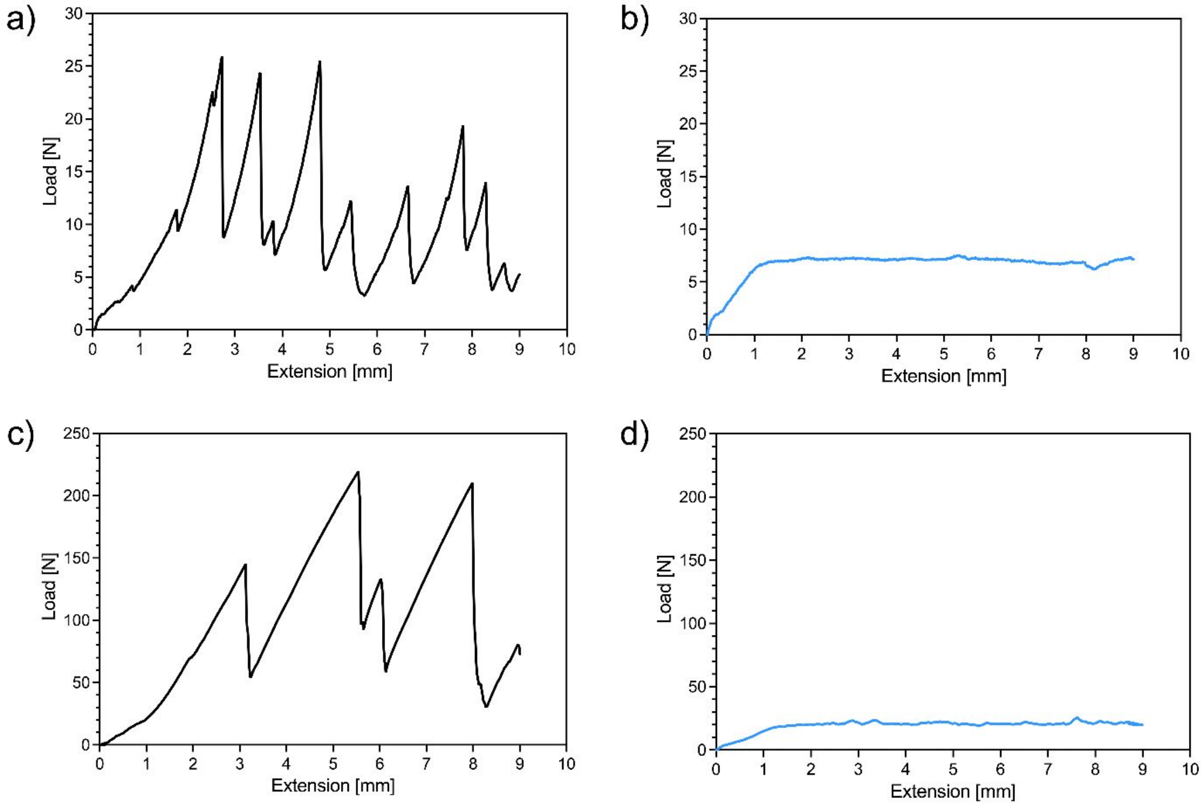

Figure S5. Extrudability test: Injection force for of chitosan and CO_2-chitosan hydrogels over extension injected from 5 mL syringe with a 18G (a,b) and 22G (c,d) needle, with a flow rate of 1 mL min^{-1}.

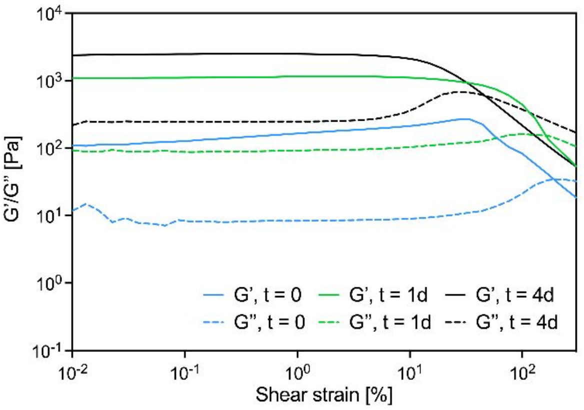

Figure S6. Degradation test: Linear viscoelastic region (LVER) evolution over time for CO_2-chitosan hydrogels incubated in cell culture environment (DMEM enriched with 1 % w w^{-1} penicillin and streptomycin and 0.02% w w^{-1} sodium azide (NaN_3)) at 37 °C for different times.

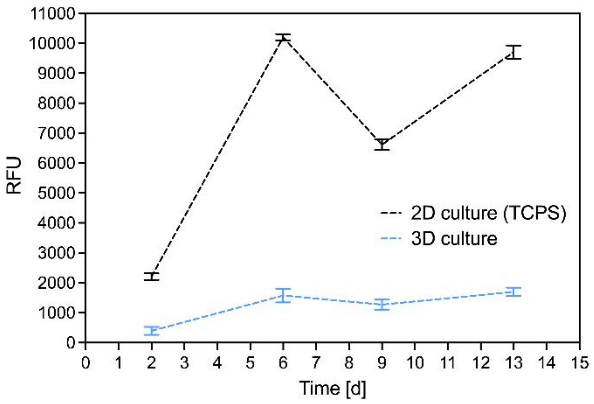

Figure S7. Direct viability at 2, 6, 9 and 13 days for pellets loaded into 2 % CO2-chitosan hydrogels (blue) and on 2D standard tissue culture plates (black).
